# Retraction: RNAi-Dependent and Independent Control of LINE1 Accumulation and Mobility in Mouse Embryonic Stem Cells

**DOI:** 10.1371/journal.pgen.1005519

**Published:** 2015-09-03

**Authors:** Constance Ciaudo, Florence Jay, Ikuhiro Okamoto, Chong-Jian Chen, Alexis Sarazin, Nicolas Servant, Emmanuel Barillot, Edith Heard, Olivier Voinnet

At the request of the authors, *PLOS Genetics* is retracting this publication following an investigation into concerns that were raised regarding the assembly of Fig 4 and S4 Fig, and the statistical analysis used in Fig 2A. The text below has been agreed to by the authors and editors.

The corresponding author, Olivier Voinnet, was originally alerted to errors that occurred during the assembly of Fig 4 (panel A) and S4 Fig (panels A and F). These errors have been corrected using the original raw data, and a correction notice was published accordingly.

Further analysis of the paper revealed flaws in the interpretation of the transposition data presented in Fig 2A. In the originally submitted version, the L1 copy number was only presented for the DCR^Flx/Flx^ P10 and DCR^-/-^ P30 cells, and a T-test performed on the two datasets showed that the L1 copy number was statistically higher in DCR^-/-^ cells than in control cells. During the last stage of the review process, additional datasets were added and a second T-test was then used to establish the statistical analysis published in the final version of the paper. However, it was later realized that T-tests are not appropriate for comparing more than two datasets. At the recommendation of the ETH statistics helpdesk, a suitable Analysis of Variance (ANOVA) test with multiple comparisons was then conducted on the Dcr^Flx/Flx^ P30 and Dcr^-/-^ P30 datasets, providing a p-value of 0.0501, which is at the margin of the threshold of significance. The ANOVA test conducted on the Dcr^Flx/Flx^ P10 and Dcr^-/-^ P30 datasets revealed a statistically significant p-value of 0.0018. The statistical issue regarding the L1 copy number in DCR^-/-^ versus control ES cells is currently being addressed using a new set of cells and a direct GFP-based transposition assay. This issue will hopefully be clarified in the near future *via* the submission of an amended study for peer-review.

Based on the present uncertainty revealed by the corrected statistical analysis of the L1 copy number—a key element of this paper—and on the previous errors in the figures, the authors have collectively decided to retract this study. Constance Ciaudo and Olivier Voinnet take full responsibility for the mistakes on this paper and wish to apologize. They also wish to state that none of the above-mentioned mistakes involved any of the co-authors from the Curie Institute, whose contributions to the paper were restricted to the bioinformatics analysis of small RNAs (NS, CJC, EB) and the generation of reagents including an ES cell line required for the study (EH, IO). All authors regret the inconvenience caused.
